# Impact of four sequential measures on the prevention of ventilator-associated pneumonia in cardiac surgery patients

**DOI:** 10.1186/cc13799

**Published:** 2014-03-26

**Authors:** María Jesús Pérez-Granda, José María Barrio, Patricia Muñoz, Javier Hortal, Cristina Rincón, Emilio Bouza

**Affiliations:** 1 Department of Anesthesiology, Hospital General Universitario Gregorio Marañón, Madrid, Spain; 2 CIBER Enfermedades Respiratorias-CIBERES (CB06/06/0058), Madrid, Spain; 3 Department of Clinical Microbiology and Infectious Diseases, Hospital General Universitario Gregorio Marañón, Madrid, Spain; 4 Department of Medicine, Universidad Complutense, Madrid, Spain; 5 Clinical Microbiology and Infectious Diseases, Hospital General Universitario Gregorio Marañón, Doctor Esquerdo, 46, 28007 Madrid, Spain

## Abstract

**Introduction:**

Ventilator-associated pneumonia (VAP) is the most frequent infection in patients admitted to intensive care units.

The efficacy of individual measures for the prevention of VAP is well documented, and data on the impact of implementing bundle measures have usually been reported from studies in which several measures are implemented simultaneously in the general intensive care unit (ICU).

The objective of our work was to evaluate the impact of four sequentially implemented measures for preventing VAP in a major heart surgery ICU. The measures were a specific training program, aspiration of subglottic secretions (ASSs), introduction of an inclinometer to improve the semirecumbent position, and reinforcement of oral care with chlorhexidine.

**Methods:**

We compared rates of VAP, days on mechanical ventilation (MV), and cost of antimicrobial agents before and during implementation.

**Results:**

We collected data from 401 patients before the intervention and from 1,534 patients during the intervention. Both groups were comparable. No significant differences in EuroSCORE were observed between the patients of both periods (6.4 versus 6.3; *P* = 0.7). The rates of VAP (episodes/1,000 days of ventilation) were, respectively, 23.9 versus 13.5 (*P* = 0.005). Mean number of days of MV/1,000 days of stay was 507 versus 375 (*P* = 0.001), and the cost of antimicrobial therapy (Euros/1,000 days of stay) was €70,612 versus €52,775 (*P* = 0.10). The main effect of sequential application of preventive measures in time achieved a relative-rate reduction of VAP of 41% (IRR, 0.41; 95% CI, 0.28 to 0.62). The mortality rate before and during the intervention was 13.0% and 10.2%, respectively.

VAP rate was most significantly reduced by training and the use of the inclinometer.

**Conclusions:**

A sequentially applied bundle of four preventive measures reduces VAP rates, days of MV, and the cost of antimicrobial therapy in patients admitted to the major heart surgery ICU.

**Trial registration:**

Clinical Trials.gov: NCT02060045. Registered 4 February 2014.

## Introduction

Ventilator-associated pneumonia (VAP) is the most frequent infection in patients admitted to the intensive care unit (ICU). It is associated with prolonged hospital stay [1-3], increased health care costs [4], and an attributable mortality ranging from 8.1% to 31.9% [5-7]. Bundles of preventive measures to reduce the incidence of VAP usually consist of interventions based on accepted guidelines [4,8,9], which are usually implemented simultaneously. They are generally evaluated in general and mixed ICUs, but not in the major heart surgery ICUs (MHS-ICU) [10].

Our objective was to evaluate the impact of four sequentially implemented measures to reduce VAP over a 35-month period in an MHS-ICU. The four measures were a specific training program, aspiration of subglottic secretions, introduction of an inclinometer to improve the semirecumbent position, and reinforcement of oral care with chlorhexidine.

## Material and methods

### Hospital setting and patients

Our institution is a general referral hospital with 1,550 beds and approximately 50,000 admissions/year. More than 500 MHS procedures are performed annually in the Department of Cardiovascular Surgery, which is a large referral unit.

### Study design

We performed an ecological prospective study with historical controls to analyze MHS patients. We compared the incidence of VAP before the bundle (9 months) and during the bundle (35 months).

The first measure was a training program provided by a panel of experts on VAP in our institution. The program consisted of eight sessions (15 minutes each) delivered to all MHS-ICU health care workers.

The second measure was systematic aspiration of subglottic secretions by using a TaperGuard Evac endotracheal tube (ETT) (Mallinckrodt, USA) over a period of 13 months. At the time of implementation of this measure, only these tracheal tubes were available in the unit to assure compliance. Tracheal aspiration through the third lumen of the ETT was performed with a negative pressure of between 100 and 150 mm Hg. Cuff pressure was maintained at between 20 and 30 mm Hg and monitored during each shift.

The third measure was the incorporation of an inclinometer in the backrest to facilitate the semirecumbent position. The fourth measure was oral care with chlorhexidine, performed every 8 hours and registered every shift. Compliance with the adequate position and oral care was measured once a day by a nurse. No other changes in patient care were included during the study period.

### Primary end point

The primary end point of the study was the reduction in the incidence density of VAP.

### Secondary end points

The secondary end points were length of ICU stay, days of MV per ICU stay, mortality rate, cost of antimicrobial acquisition during ICU stay, and compliance with the measures.

### Ethics

The Ethics Committee of our institution (Hospital Gregorio Marañon) approved the study and waived the need for informed consent because we follow the recommendations of the guidelines for the prevention of ventilator-associated pneumonia.

### Follow-up

Physicians from the Departments of Anesthesia and Infectious Diseases monitored patients daily to check for the presence of infections. The infection-control team is multidisciplinary and comprises physicians and nurses from the ICU, microbiologists, infectious diseases specialists, and health care workers from the Preventive Medicine Department. Data were collected systematically on a preestablished data form that is routinely used in the postsurgical MHS-ICU.

### Sampling in patients with suspected lower respiratory tract infection

Sampling of the lower respiratory tract in patients with suspected VAP was by endotracheal aspiration, protected specimen brushing, or both. When aspiration was unproductive, we irrigated with 5 ml of Ringer lactate solution. Secretions obtained by endotracheal aspiration were collected in a Lukens specimen container (Sherwood Medical, Tullamore, Ireland). A sample was considered positive with bacterial counts ≥10^4^ cfu/ml for each microorganism obtained by using endotracheal aspiration and ≥10^3^ cfu/ml for each microorganism obtained by using protected specimen brushing.

All microorganisms were identified by using standard methods, and antimicrobial susceptibility was determined according to Clinical and Laboratory Standards Institute (CLSI) recommendations.

### Demonstration of VAP

Patients ventilated for >48 hours were diagnosed with VAP based on the presence of new and/or progressive pulmonary infiltrates on the chest radiograph plus two or more of the following criteria: fever >38.5°C or hypothermia <36°C, leukocytosis ≥12 × 10^9^/L, purulent tracheobronchial secretions, and a ≥15% reduction in PaO_2_/FiO_2_, according to the definitions of the Centers for Disease Control and Prevention [11]. Patients with a clinical pulmonary infection score (CPIS) higher than 6 were also considered to have pneumonia [12]. The isolation of one or more pathogenic microorganisms in significant bacterial counts was required to confirm the diagnosis of VAP.

Unless other evidence was available, we considered as nonpathogenic the isolation (at any concentration) of the following microorganisms in lower respiratory secretions: viridans-group streptococci, coagulase-negative staphylococci, *Neisseria* spp, *Corynebacterium* spp, and *Candida* spp.

Diagnosis practices did not change after training, and no surveillance cultures were regularly performed in the unit.

### Statistical analysis

Relations between baseline variables were evaluated before and during implementation of the different preventive measures. Baseline comparisons between groups were established by clinical relevance. The qualitative variables appear with their frequency distribution. The quantitative variables are summarized as the mean and standard deviation (SD) or median with IQR, if necessary. Continuous variables were compared by using the *t* test for normally distributed variables or median test for nonnormally distributed variables. The χ^2^ or Fisher Exact test was used to compare categoric variables.

The incidence rates of respiratory tract infection (pneumonia) (event/1,000 days of MV), the antimicrobial cost, and the days of mechanical ventilation were compared before and during the interventions. To evaluate the impact of sequential measures, we performed a time-series analysis with a nonsegmented Poisson regression test. The change in the temporal trend was expressed as incidence rate ratio (IRR) and 95% confidence interval (CI) for the whole model and for each sequential measure. The IRR expresses the accumulative effect of each intervention implemented so far.

The slopes for days of mechanical ventilation, days of ICU stay, and cost of antimicrobial agents were calculated with a lineal regression model.

All statistical tests were two-tailed. Statistical significance was set at *P* < 0.05 for all the tests. The statistical analysis was performed with SPSS 12.0 and Stata 11.0.

## Results

We compared the results obtained before the interventions (November 2008 to July 2009) and during the interventions (August 2009 to June 2012).

The underlying conditions and characteristics of the populations included before and after the interventions are compared in Table 1. No significant differences in the underlying conditions and situation of the populations in either period were detected, and both groups were comparable.

**Table 1:** Baseline characteristics and surgical variables of study patients

	**Before implementation of bundle**	**During implementation of bundle**	***P *** **value**
	***n*** **= 401**	***n*** **= 1,534**	
**Preoperative**			
Mean age in years (SD)	66.45 (12.0)	67.36 (30.6)	0.56
Male sex F/M	162/635	239/897	0.88
Underlying conditions (%)			
Myocardial infarction	59 (14.7)	173 (10.4)	0.06
Congestive heart failure	65 (16.2)	217 (14.1)	0.26
Central nervous system disease	18 (4.5)	111 (7.2)	0.05
Chronic obstructive pulmonary disease	71 (17.7)	227 (14.8)	0.15
Renal dysfunction	17 (3.7)	47 (2.82)	0.24
Diabetes mellitus	111 (27.7)	419 (27.3)	0.42
Peptic ulcer disease	11 (2.7)	40 (2.6)	0.88
Peripheral vascular disease	30 (7.5)	118 (7.7)	0.88
Euroscore (±SD)	7.30 (3.33)	6.86 (3.83)	0.93
Severe pulmonary hypertension (%)	60 (15.0)	217 (14.1)	0.67
**Type of surgery** (%)			
Valve replacement	190 (47.4)	696 (45.4)	0.47
CABG	89 (22.2)	362 ( 23.6)	0.55
Mixed (valve and CABG)	58 (14.5)	191 (12.5)	0.28
Aortic surgery	25 (6.2)	114 (7.4)	0.97
**Operative data**			
Mean CPBT (min) (SD)	115.2 (64.0)	118.1 (67.6)	0.92
Mean aortic cross-clamp time (min) (SD)	70.5 (35.0)	76.6 (37.6)	0.18
Intraaortic balloon during the surgery	49 (12.2)	164 (10.7)	0.38

Type of surgery, mean time on cardiopulmonary bypass, aortic cross-clamp time, and other data obtained during surgery were similar (Table 1).

### Primary end point: incidence of VAP

**Figure 1:**
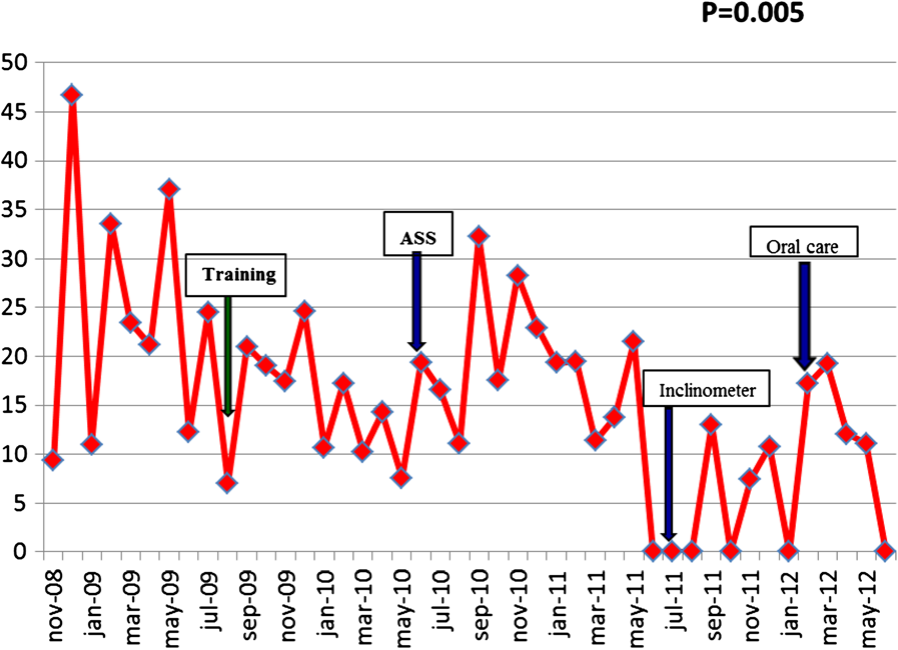
Incidence density of VAP during the study period.

The rates of VAP before and during the intervention were 23.92/1,000 days and 13.49 episodes/1,000 days of MV, respectively (*P* = 0.005) (Figure 1 and Table 2). To check the stability of the preintervention figures, we obtained data from January 2007. The incidence density of VAP in the MHS-ICU during that period was 22.94 episodes/1,000 days of MV. We did not observe any significant differences in the proportion of etiologic agents of VAP between the two periods (Table 3). The accumulated monthly effect of each intervention individually and as a bundle is showed in Table 4. The IRR of VAP decreased by 51% after the implementation of the training program (IRR, 0.51; 95% CI, 0.34 to 0.78) and after the introduction of the first three measures, VAP decreased another monthly 45% (IRR, 0.45; 95% CI, 0.24 to.84).

**Table 2:** Primary and secondary end points

	**Before (9 mo)**	**Training (10 mo)**	**AAS (13 mo)**	**Inclinometer (7 mo)**	**Oral care (5 mo)**	**During (35 mo)**	***P *** **value**
	**Pts: 401**	**Pts: 453**	**Pts: 545**	**Pts: 294**	**Pts: 242**	**Pts: 1,534**	
Median length of ICU stay in days (IQR)	4 (2-7)	5 (3-7)	4 (3-6.5)	4 (2-7)	4 (3-6)	4 (3-7)	0.47
Median days on MV (IQR)	1 (1-1)	1 (1-1)	1 (1-1)	1 (1-1)	1 (1-1)	1 (1-1)	0.37
Mean days on VM (SD)	4.1 (11.9)	2.7 (5.7)	3.1 (8.9)	2.4 (4.6)	3.1 (9.1)	2.8 (7.4)	0.05
VAP/1,000 days of MV	23.9	14.8	17.8	4.8	10.9	13.5	0.005
Mean days of MV/1,000 days of stay (SD)	507 (128)	412 (103)	359 (85)	342 (99)	390 (77)	375 (93)	0.001
Primary cost of antimicrobial/1,000 days of stay	€70,612	€94,839	€39,564	€30,153	€34,671	€52,775	0.10
Mortality (%)	52 (13.0)	56 (10.4)	56 (10.3)	21 (7.1)	23 (9.5)	156 (10.2)	0.10

MV, mechanical ventilation, Pts, patients.

**Table 3:** Distribution of microorganisms isolated in the episodes of ventilator-associated pneumonia before and during the bundle

	**Before BUNDLE**	**During BUNDLE**	***P***
	**40**	**62**	
Methicillin-resistant *Staphylococcus aureus*	3	1	0.29
Methicillin-susceptible *Staphylococcus aureus*	2	3	>0.99
Other Gram-positive microorganisms	2	5	0.70
Enterobacteriaceae	18	33	0.54
*Pseudomonas aeruginosa*	15	19	0.52
*Acinetobacter* spp.	0	1	>0.99
*Stenotrophomonas maltophilia*	3	4	>0.99
*Haemophilus influenzae*	2	1	0.55

**Table 4:** Time-series analysis of the accumulated monthly effect of each intervention individually and of the whole bundle of measures

		**Beta**	**95% CI Beta**		***P***
VAP rate*		Mean change by month			
Full model	Before and after training	0.50	0.29	0.84	0.009
	Before and after ASS	1.05	0.60	1.85	0.856
	Before and after inclinometer	0.26	0.09	0.74	0.011
	Before and after oral care	0.02	0.01	0.03	0.117
Final model	Before and after training	0.51	0.34	0.78	0.002
	Before and after inclinometer	0.45	0.24	0.84	0.013
	Bundle	0.41	0.28	0.62	<0.001
Cost of antimicrobial/1,000 days of stay					
Full model	(Constant)	56,800	29.84	83.77	0.000
	Before and after training	-2.010	-45.11	41.09	0.925
	Before and after ASS	-87.040	-133.81	-40.26	0.001
	Before and after inclinometer	-37.030	-81.77	7.71	0.102
	Before and after oral care	-12.060	-54.31	30.20	0.567
	Monthly change	2.760	-0.58	6.10	0.102
Final model	(Constant)	59,110	33.93	84.30	0.000
	Before and after ASS	-82,600	-122.64	-42.55	0.000
	Before and after inclinometer	-37,840	-73.79	-1.90	0.040
	Monthly change	2,420	0.35	4.50	0.023
	Bundle	-17,840	-47.59	11.92	0.233
Days of MV/1,000 days of stay					
Full model	(Constant)	489.47	402.19	576.75	0.000
	Before and after training	-128.51	-268.02	11.00	0.070
	Before and after ASS	-93.99	-245.38	57.39	0.216
	Before and after inclinometer	-51.81	-196.61	92.99	0.473
	Before and after oral care	26.75	-110.01	163.52	0.694
	Monthly change	3.54	-7.26	14.34	0.511
Final model	(Constant)	507.17	439.70	574.65	0.000
	Before and after training	-131.70	-207.35	-56.04	0.001
	Bundle	-131.70	-207.35	-56.04	0.001

*Data was expressed as incidence rate ratio by Poisson model.

### Secondary end points

#### Cost of antimicrobial acquisition during ICU stay

**Figure 2:**
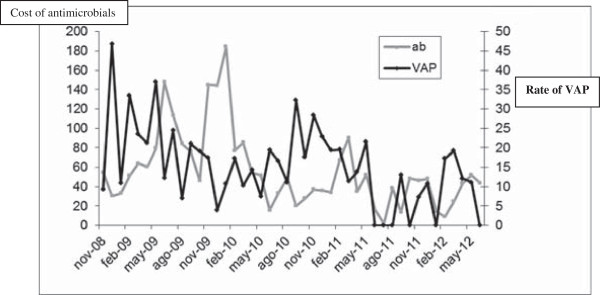
Evolution of cost of antimicrobials and VAP rate during study period.

The cost of acquisition of antibiotics fell between the two periods (€70,612/1,000 days of ICU stay versus €52,775/1,000 days of ICU stay; *P* = 0.10) (Table 2). Table 4 and Figure 2 show the accumulated monthly effect of each intervention in antimicrobial cost.

#### Days of MV per ICU stay

**Figure 3:**
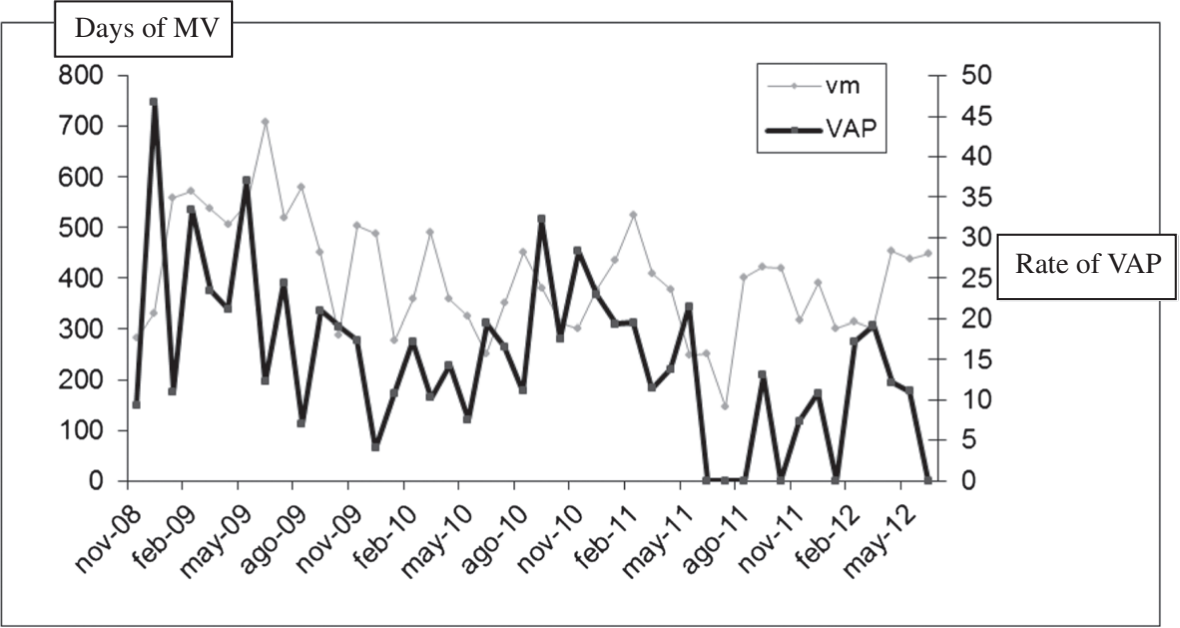
Evolution of MV/1,000 days of ICU stay and VAP rate during the period of study.

The mean number of days of MV/1,000 days of stay was estimated. Mean days of MV/1,000 days of ICU stay before and after the intervention were, respectively, 507/1,000 days of stay and 375/1,000 days of stay (*P* = 0.001) (Figure 3 and Table 2). The overall reduction in the mean number of days on MV was 131.70 MV/1,000 days of stay (95% CI, -207.35 to -56.04) (Table 4). Training was the only measure with a significant effect on the days of MV.

#### Compliance with the measures

The degree of compliance with the education, systematic aspiration of subglottic secretions, and oral care with chlorhexidine every 8 hours was 100%. However, it was lower for the implementation of the ideal semirecumbent position to an adequate angle (between 30 degrees and 45 degrees). It was only slightly modified from one period to the next with the inclusion of the inclinometer (mean before, 40.26% versus mean after, 42.05%; *P* = 0.68).

The mortality rate before and after the intervention was 13% (52 patients) and 10.2% (156 patients), *P* = 0.10.

## Discussion

The sequential implementation of a bundle of measures to prevent VAP in patients undergoing MHS reduced the incidence density of VAP and the days on MV. A trend was noted to the reduction of the cost of acquisition of antimicrobial agents.

VAP is the most frequent infection after MHS, with incidence rates ranging from 5.7% to 21.6% and incidence densities ranging from 22.2/1,000 days of MV to 34.5/1,000 days of MV in all patients undergoing surgery [2,13-15].

VAP is associated with a high mortality rate, but only a few predisposing risk factors can be modified [16-18]. Many groups and scientific societies have provided prevention guidelines in the last 10 years [19-22]. Several guidelines recommend different measures to decrease the incidence of VAP, including training, which quickly reduces the incidence of VAP rates, although its long-term efficacy is limited [23,24].

Pathogenic mechanism of VAP is mainly by aspiration of secretions with bacteria colonizing the upper respiratory tract and passing into the lower respiratory tract via the leaks between the tracheal wall and the cuff of the endotracheal tube (ETT). The use of ETTs with a third lumen that permits aspiration of subglottic secretions has been associated with a reduction in VAP rates [25]. Despite being recommended by guidelines, this measure is far from being universally implemented in ICUs [2].

A backrest elevation of 30 degrees to 45 degrees is recommended to decrease the incidence of VAP, although implementation is influenced by clinical practice and the patient’s condition, and subjective perception of the angle of inclination is limited [26-29].

Oral care also plays a role in the prevention of VAP, although no uniform protocol of application is currently available [30,31].

Many ICUs cannot implement simultaneously all the measures included in a bundle. Our study shows that sequential introduction of preventive techniques have a global impact in the reduction of VAP, but the design of our study, with measures that are accumulated additively to the previous ones, do not permit clearly an estimation of the impact of simple individual measures.

Our study is limited in that we enrolled only the population undergoing MHS; therefore, our data cannot necessarily be extrapolated to other populations.

## Conclusion

Our study shows that the implementation of preventive measures for VAP are effective, even if the measures were implemented in the high-risk population for VAP admitted into Major Heart Surgery ICUs.

## Key messages

• A prevention of VAP in MHS-ICUs is feasible with the implementation of four simple measures, even when sequentially implanted.

## Abbreviations

ICU: Intensive care unit; MHS: major heart surgery; MV: mechanical ventilation; SD: standard deviation; VAP: ventilator-associated pneumonia.

## Competing interests

The authors declare that they have no competing interests.

## Authors’ contributions

EB, MJPG, and PM: study conception and design; data acquisition, analysis, and interpretation; manuscript writing, and final approval of the manuscript. JMB, JH, and CR: data acquisition and analysis, critical revision, and final approval of the manuscript. All authors read and approved the final manuscript.
